# Silver nanoparticles induce the cardiomyogenic differentiation of bone marrow derived mesenchymal stem cells via telomere length extension

**DOI:** 10.3762/bjnano.12.62

**Published:** 2021-08-02

**Authors:** Khosro Adibkia, Ali Ehsani, Asma Jodaei, Ezzatollah Fathi, Raheleh Farahzadi, Mohammad Barzegar-Jalali

**Affiliations:** 1 Research Center for Pharmaceutical Nanotechnology, Tabriz University of Medical Sciences, Tabriz, Iran; 2 Department of Pharmaceutical Sciences, Faculty of Pharmacy, Tabriz University of Medical Sciences, Tabriz, Iran; 3 Student Research Committee, Tabriz University of Medical Sciences, Tabriz, Iran; 4 Department of Clinical Sciences, Faculty of Veterinary Medicine, University of Tabriz, Tabriz, Iran; 5 Hematology and Oncology Research Center, Tabriz University of Medical Sciences, Tabriz, Iran; 6 Pharmaceutical Analysis Research Center, Tabriz University of Medical Sciences, Tabriz, Iran

**Keywords:** bone marrow-derived MSCs, cardiomyogenic differentiation, silver nanoparticles, telomere length, Wnt3/β-catenin signaling pathway

## Abstract

Finding new strategies for the treatment of heart failures using stem cells has attracted a lot of attention. Meanwhile, nanotechnology-based approaches to regenerative medicine hypothesize a possible combination of stem cells and nanotechnology in the treatment of diseases. This study aims to investigate the in vitro effect of silver nanoparticles (Ag-NPs) on the cardiomyogenic differentiation of bone marrow-derived mesenchymal stem cells (BM-MSCs) through detection of cardiac markers. For this purpose, MSCs were isolated from bone marrow resident and differentiated to the cardiac cells using a dedicated medium with Ag-NPs. Also, the cardiomyogenic differentiation of BM-MSCs was confirmed using immunocytochemistry. Then, real-time PCR and western blotting assay were used for measuring absolute telomere length (TL) measurement, and gene and protein assessment of the cells, respectively. It was found that 2.5 µg/mL Ag-NPs caused elongation of the telomeres and altered VEGF, C-TnI, VWF, SMA, GATA-4, TERT, and cyclin D protein and gene expression in the cardiomyogenically differentiated BM-MSCs. Also, there was a significant increase in the protein and gene expression of Wnt3 and β-catenin as main components of pathways. We concluded that Ag-NPs could change the in vitro expression of cardiac markers of BM-MSCs via the Wnt3/β-catenin signaling pathway.

## Introduction

Cardiac disorders that eventually lead to heart failure cause an increased loss of cardiac cells. There is strong evidence that the progression of heart failure is associated with reduction in the number of myocytes due to myocyte necrosis and apoptosis [1]. In the therapies of chronic heart failure (CHF) adrenergic and angiotensin signaling pathways are interrupted, while the therapies do not replace the dying myocytes during CHF progression [2]. Stem cells and some progenitor cells have recently gained much attention in cell therapy regarding the repair of damaged heart tissue [3]. In regenerative medicine, bone marrow mesenchymal stem cells (BM-MSCs) and cardiac progenitor cells play a remarkable role in the regeneration of the myocardium [4]. Experimental studies related to the role of MSCs have been done in the form of animal studies as well as clinical trials [5–6]. Nevertheless, injection of these cells to the heart tissue causes poor differentiation into specialized cardiac cells, which is associated with rapid destruction of the engrafted cells. With the definitive goal of expediting stem cell therapy [7], scientists are trying hard to facilitate the differentiation of MSCs into other types of cardiac cells, including endothelial or smooth muscle cells, in vitro. Meanwhile, the use of 3-dimensional (3D) culture and nanomaterials for cell survival and preservation has attracted attention.

Novel nanomaterials are being developed to improve disease treatment processes via biopharmaceutical molecules as well as the surface treatment of biomaterials [8–9]. Among nanoparticles (NPs), silver nanoparticles (Ag-NPs) are successfully commercialized due to their well-known antiseptic properties. Several studies have used different types of cell lines under in vitro conditions to investigate the properties, differential responses, and mechanisms of action of Ag-NPs [10]. Sengstock et al. showed that Ag-NPs attenuate the adipogenic and osteogenic differentiation of human MSCs. These inhibitory effects were shown through a decrease in the secretion of specific biomarkers, including adiponectin (adipocytes) and osteocalcin (osteoblasts) [11]. In another study, it was indicated that Ag-NPs changed the cell morphology of mouse embryonic stem cells (mESCs). Cell cycle analysis demonstrated that Ag-NPs induced mESCs cell cycle arrest at the G1 and S phases through inhibition of the hyperphosphorylation of Retinoblastoma protein [12]. Kalishwaralal et al. reported that Ag-NPs could inhibit cell proliferation, cell viability, and cell migration through activating caspase-3 and suppressing Akt phosphorylation in bovine endothelial cells [13]. It has been determined that the positive and negative effects of Ag-NPs entirely depend on size, time point, and cell type. Both positive and negative impacts of Ag-NPs on stem cell differentiation were previously reported by studies. In this regard, the influence of Ag-NPs was demonstrated on multi-lineage differentiation of MSCs such as osteogenic, adipogenic, and neurogenic types.

Other factors, including telomeres, also influence differentiation. Studies using telomerase inhibitors and telomerase reverse transcriptase (TERT) antisense technology indicate that telomerase maintains cells in a proliferative state, and its downregulation parallels cellular differentiation [14]. Telomerase is an enzyme responsible for maintaining the telomeric repetition at the end of the chromosomes. Different cell lines have different telomere lengths (TL) [15]. Previous reports have shown that telomerase activity diminished when cells are exposed to stimuli that inhibit cell proliferation and promote differentiation [16]. Studies on the role of telomeres and telomerase in cardiac cells, cardiac differentiation, and treatment of cardiovascular diseases have also been reported. Following myocardial infarction, telomerase expression in cardiomyocytes increases significantly, implying that telomerase plays a role in regulating tissue repair in heart diseases [17]. It was pointed that critically shortened telomeres activate a series of downstream changes that induce cardiomyocyte cell cycle arrest and cellular senescence. Hence, finding solutions to increase the TL of cardiac cells could be helpful. We hypothesize that Ag-NPs can increase the cardiomyogenic differentiation potential of BM-MSCs. Also, it seems that Ag-NPs could change the cardiac differentiation of BM-MSCs via TL extension as an effective factor in reducing cell senescence of BM-MSCs.

## Results

### Multilineage differentiation of BM-MSCs

**Figure 1:**
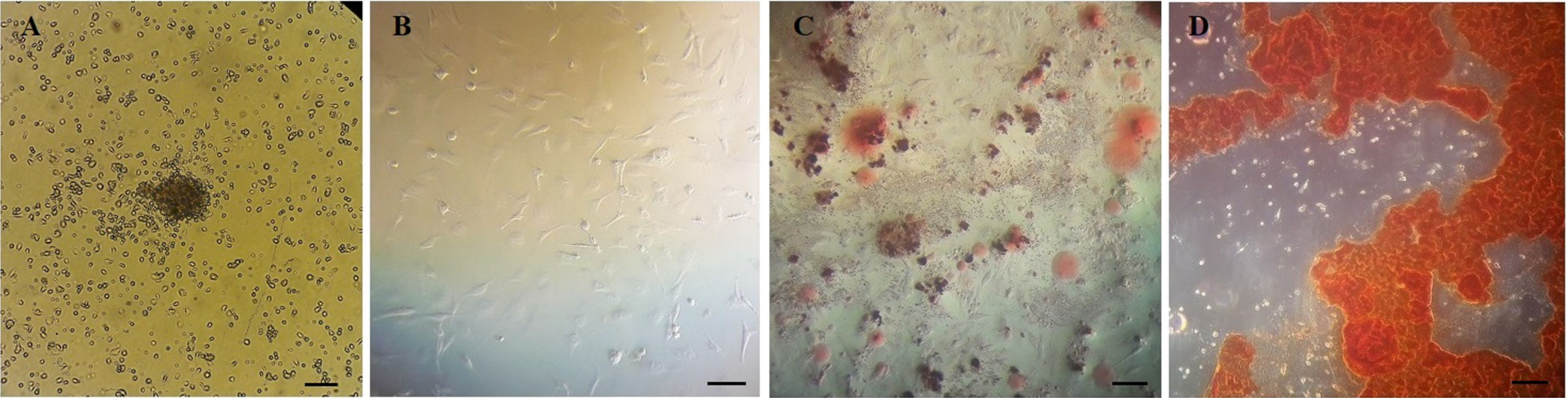
Morphology of BM-MSCs; (A) Fibroblast-like morphology of cells that appear 24 h after cell seeding (bar = 20 μm); (B) more confluent cells at the end of seven days of culture (bar = 20 μm); (C) lipid vacuoles were stained by Oil-Red O after adipogenesis (bar = 20 μm); (D) mineralized cell aggregates was stained by Alizarin red at the end of osteogenic differentiation (bar = 20 μm).

The BM-MSCs appeared as spindle-shaped cells (Figure 1A,B). The adipogenic and osteogenic differentiation was specified by Oil red O and Alizarin red staining, respectively. After staining, the presence of oil vacuoles confirmed the adipogenic differentiation (Figure 1C). Also, the redness of the nodules indicated the presence of mineralized compartments as a result of the osteogenic confirmation (Figure 1D).

### Phenotypical characterization of BM-MSCs

**Figure 2:**
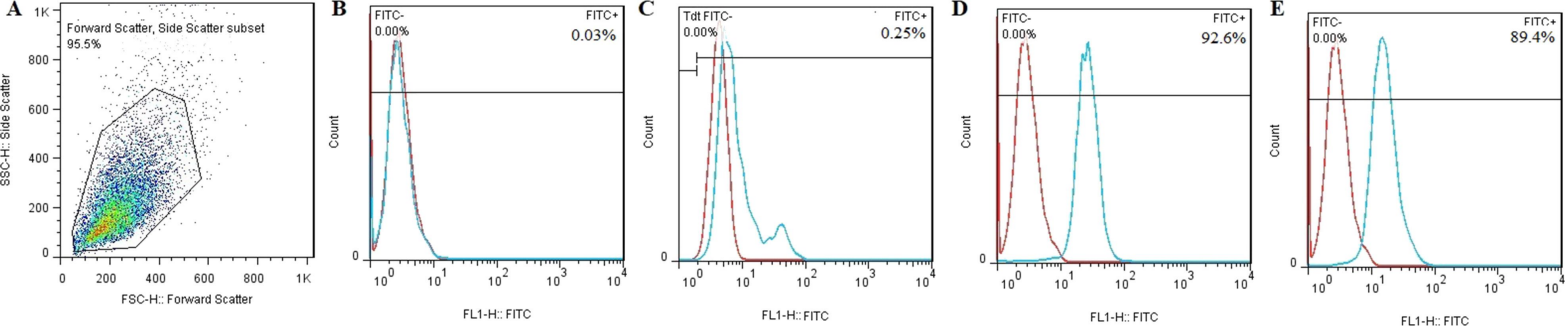
Phenotypical characterization of BM-MSCs by flow cytometry. (A) The cell population; The BM-MSCs were negative for (B) CD31 (0.03%) and (C) CD34 (0.25%) and positive for (D) CD44 (92.6%) and (E) CD90 (89.4%). Flow cytometry data was analyzed by FlowJo software (version 6.2).

Flow cytometry was carried out for the immunophenotypic characterization of BM-MSCs. The mesenchymal cell surface markers CD44 and CD90 as positive markers and the hematopoietic cell surface markers CD31 and CD34 as negative markers were investigated. As shown in Figure 2A–E, the BM-MSCs were negative for CD31 (0.03%) and CD34 (0.25%) and positive for CD44 (92.6%) and CD90 (89.4%).

### Cardiomyogenic differentiation confirmation of BM-MSCs

**Figure 3:**
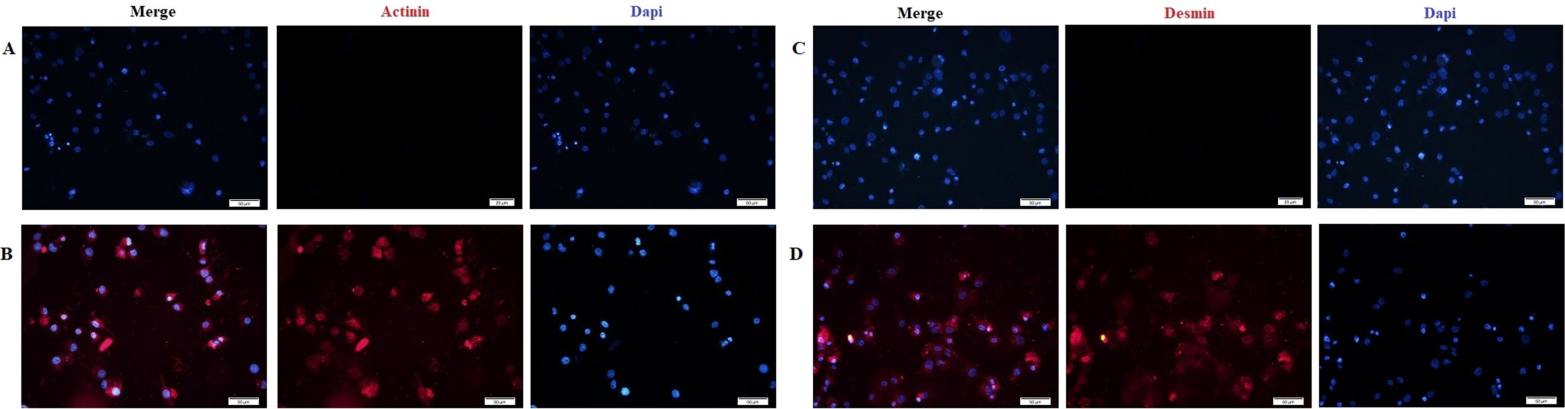
BM-MSCs differentiate into the cardiac lineage in vitro. A and C are negative controls (BM-MSCs cells in the absence of cardiac differentiation culture medium); (B) actinin (red)-positive and (D) desmin (red)-positive cells expressed in the cardiomyogenically differentiated BM-MSCs; nuclei were stained by DAPI in blue.

According to previous studies, MSCs can differentiate into cardiomyocyte cells if they are cultured in a specific cardiomyocyte differentiation medium [18–19]. In this section, the cardiomyogenic differentiation was confirmed via immunocytochemistry (ICC). Briefly, as shown in Figure 3A–D, when the BM-MSCs were cultured in the cardiomyocyte differentiation medium for a period of 14 days, they exhibited the cardiac markers of α-actinin and desmin.

### Cell proliferation assay

**Figure 4:**
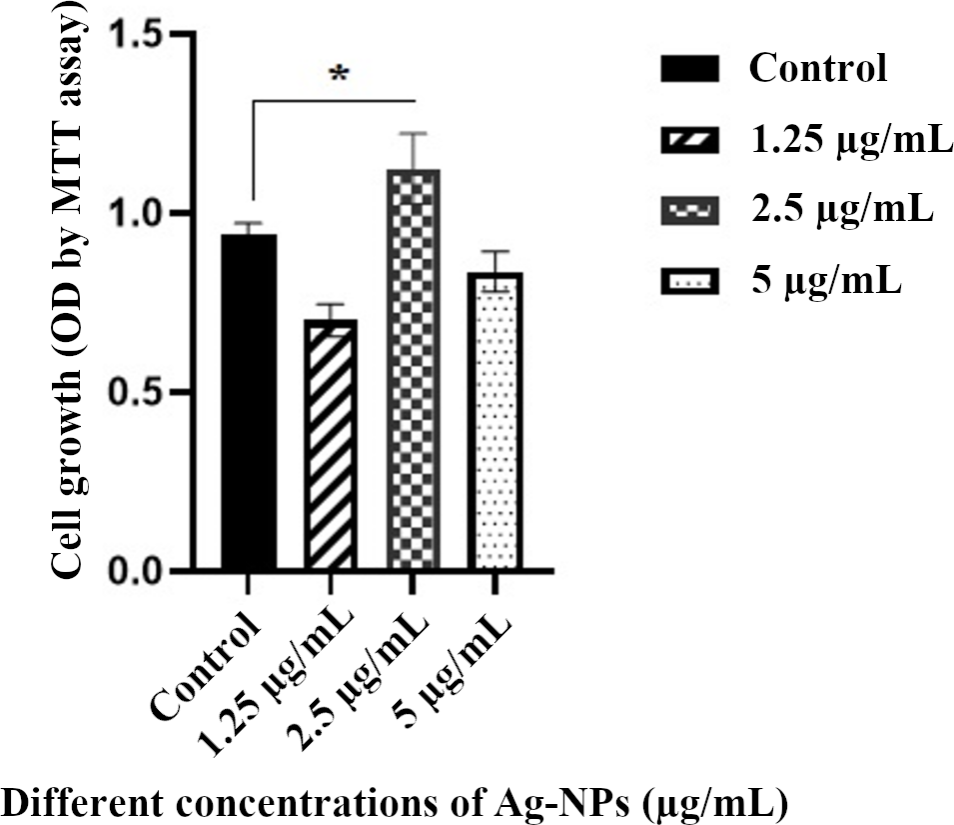
Proliferation rates of cardiomyogenically differentiated BM-MSCs in the presence of different concentrations of Ag-NPs for 14 days, using MTT assay (*p < 0.05 compared with control group).

Cardiomyogenically differentiated BM-MSCs were treated with different concentrations of Ag-NPs for 14 days and cell proliferation was examined by MTT assay. As shown in Figure 4, there was a significant difference in the proliferation of cardiomyogenically differentiated BM-MSCs between the treated groups with 2.5 µg/mL of Ag-NPs and the control group (*p < 0.05). For this reason, a concentration of 2.5 µg/mL of Ag-NPs was selected for cell treatment. Also, this concentration was previously reported by Sengstock and co-workers [11].

### Ag-NPs increase the absolute telomere length (aTL)

**Figure 5:**
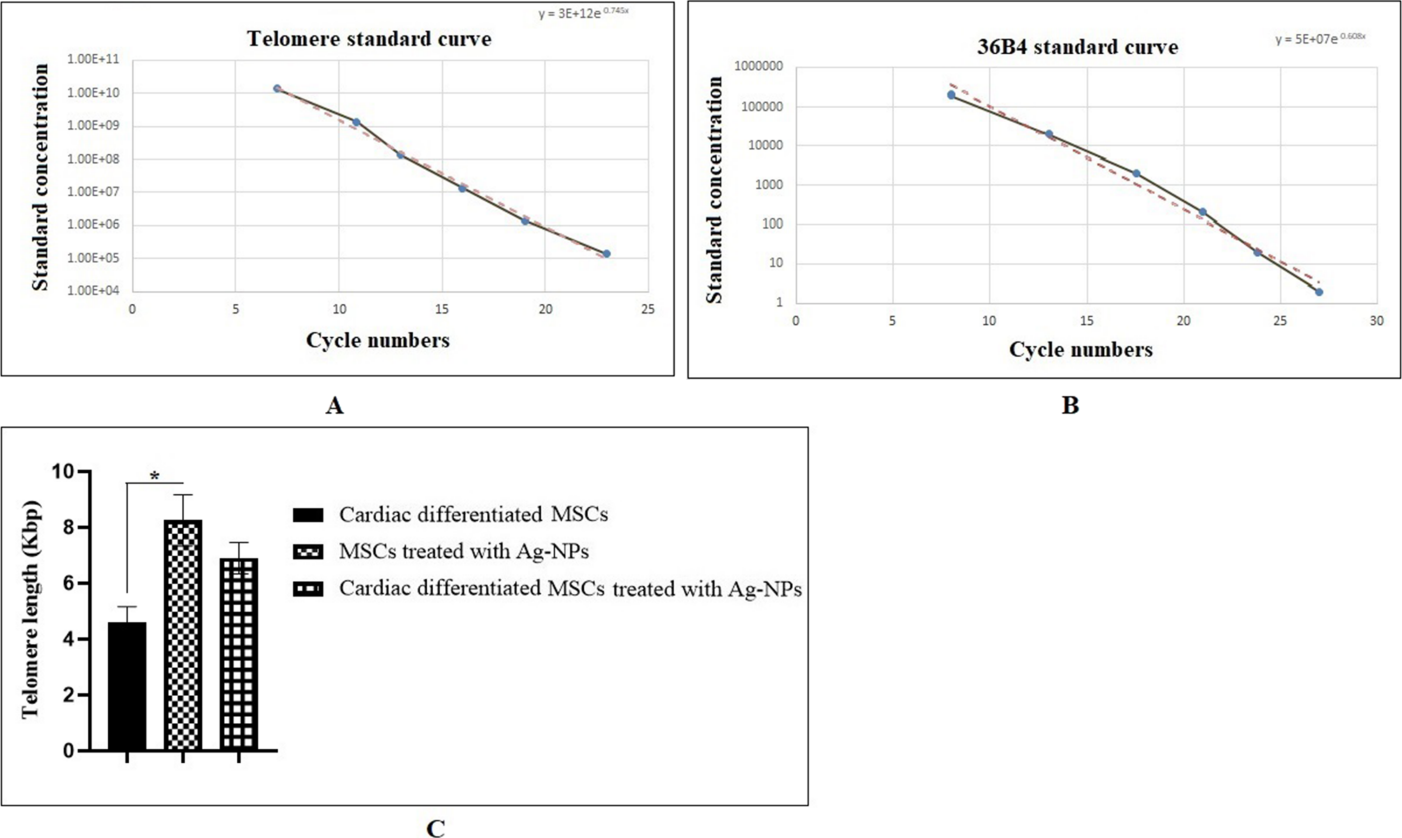
aTL measurement of cardiomyogenically differentiated BM-MSCs. (A) Standard curve for calculating the aTL sequence per reaction tube; (B) standard curve for calculating genome copies using the 36B4 copy number (The *x*-axis represents the number of the cycle and the *y*-axis shows the concentration of the standard). (C) Real-time PCR was carried out with 20 ng/μL of DNA from three groups for evaluating the aTL in triplicate (*p < 0.05; compared with control group, *n* = 3). The data were analyzed as kb/reaction and the genome copies/reaction for the telomere and the SCG.

The aTL was measured by the quantitative real-time PCR. After incubation of the BM-MSCs in the presence of Ag-NPs, genomic DNA was isolated from experimental groups I (cardiomyogenically differentiated BM-MSCs without Ag-NP treatment), II (BM-MSCs treated with Ag-NPs), and III (cardiomyogenically differentiated BM-MSCs treated with Ag-NPs), and real-time PCR was carried out. Two standard curves 4A and 4B were used to calculate the aTL. As shown in Figure 5C, the aTL was increased in group II (8.25 kbp) and III (6.90 kbp) compared to the control group (4.5 kbp). This increase was only significant in group II (*p < 0.05).

### Ag-NPs increase the protein and gene expression of TERT and cyclin D

**Figure 6:**
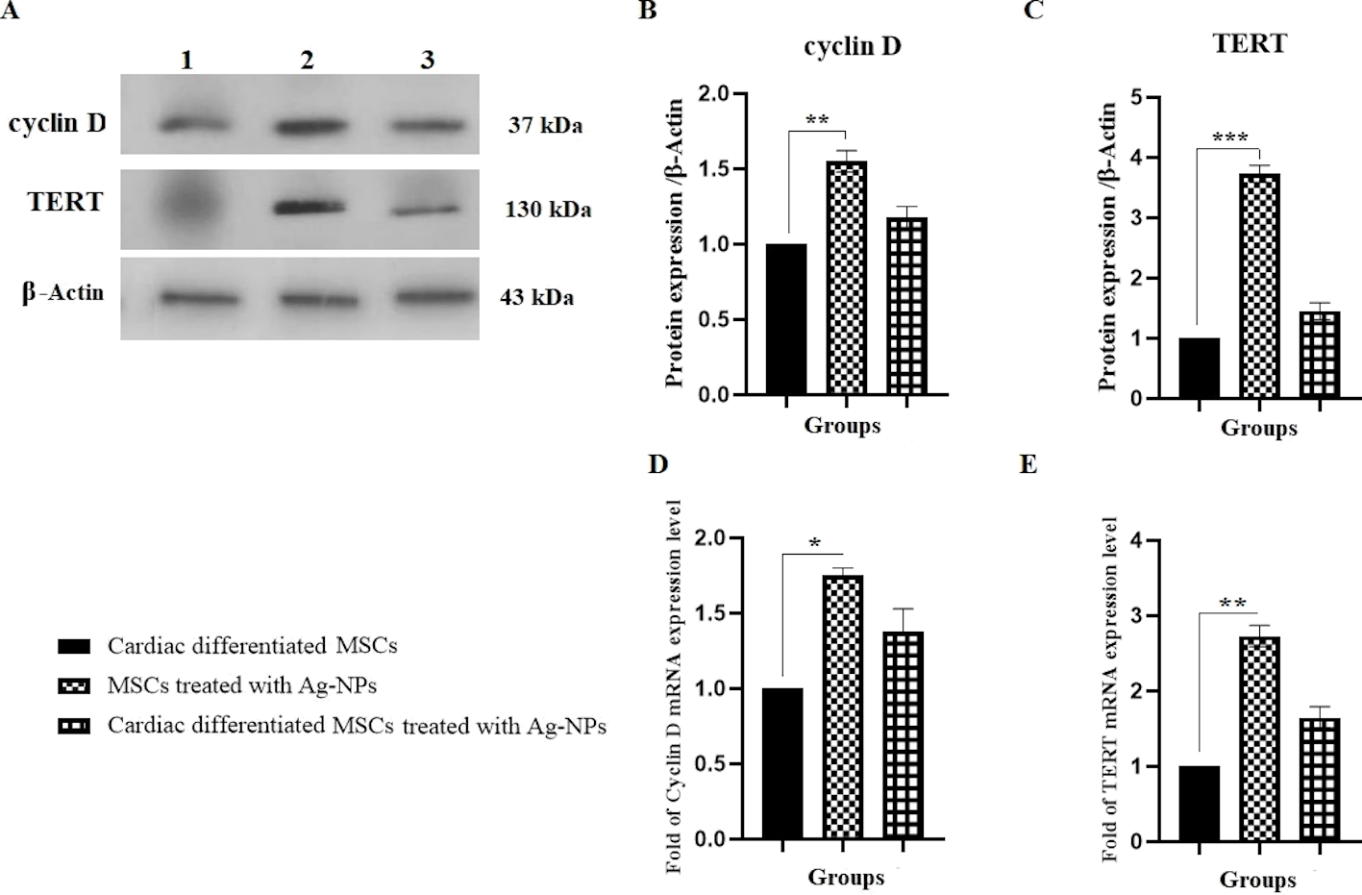
Effect of Ag-NPs on TERT and cyclin D1 protein and gene expression in cardiomyogenically differentiated BM-MSCs. Cells were cultured in 6-well plates at a concentration of 1 × 10^6^ cells/well. Protein and RNA were extracted from group I (cardiomyogenically differentiated BM-MSCs without Ag-NP treatment), group II (BM-MSCs in the presence of 2.5 µg/mL Ag-NPs), and group III (cardiomyogenically differentiated BM-MSCs in the presence of 2.5 µg/mL Ag-NPs) as described in the Experimental section and used for western blotting (A–C); (**P < 0.01 and ***P < 0.001), and real-time PCR (D and E); (*P < 0.05 and **P < 0.01), respectively.

The effect of Ag-NPs on TERT protein and gene expression as telomerase catalytic subunit was investigated using western blotting and real-time PCR, respectively. Also, the protein and gene expression of cyclin D as cell cycle checkpoint was evaluated. The protein expression levels of TERT and cyclin D1 significantly increased in group II (BM-MSCs with Ag-NPs), as compared to group I (cardiomyogenically differentiated BM-MSCs without Ag-NP treatment), by factors of about 3.72 and 1.65, respectively. A non-significant increase in the protein expression levels of TERT and cyclin D1 was also observed in group III (cardiomyogenically differentiated BM-MSCs with Ag-NPs) as compared to group I by factors of about 1.45 and 1.18, respectively (Figure 6A–C). As shown in Figure 6D,E, the mRNA expressions of cyclin D and TERT have significantly increased in group II as compared to group I by factors of about 1.75 and 1.38, respectively.

### Induction of protein and gene expression of cardiac markers

**Figure 7:**
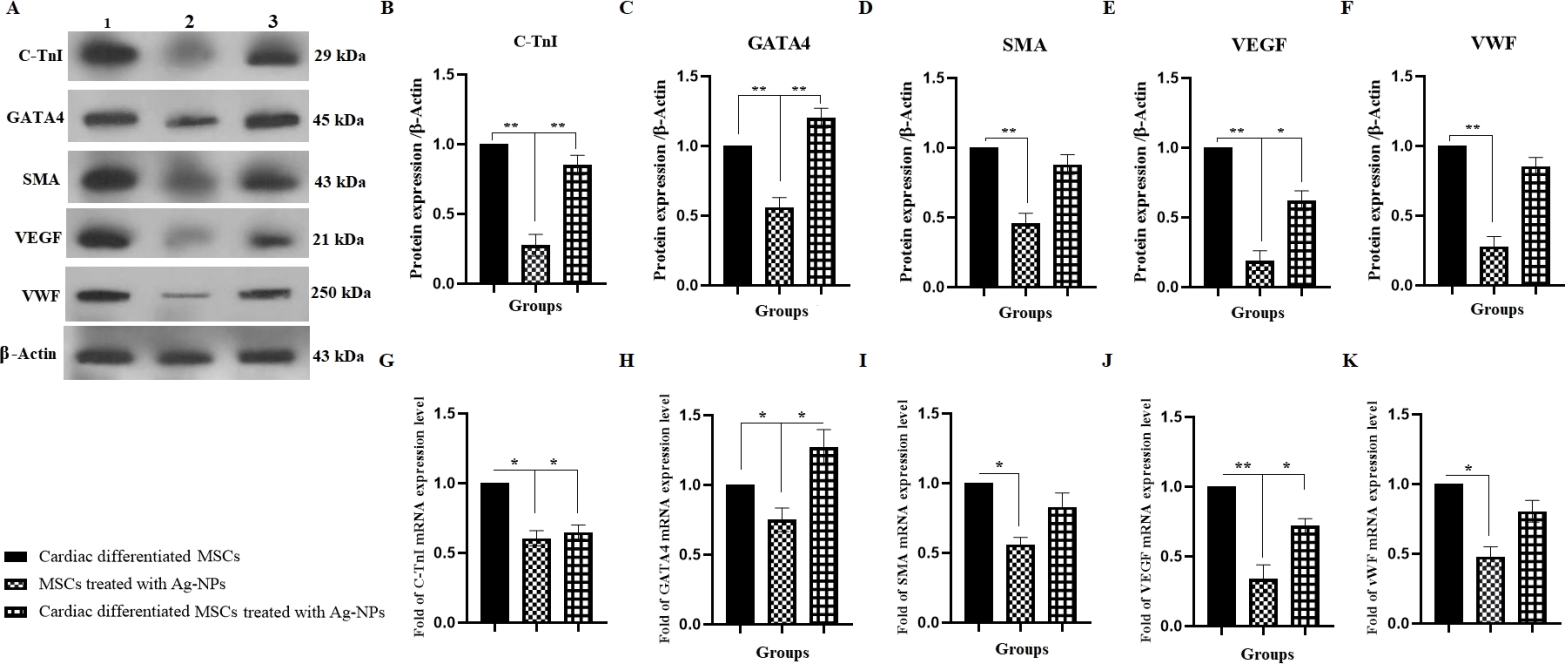
Effect of Ag-NPs on C-TnI, GATA4, SMA, VEGF, and VWF protein and gene expression in cardiomyogenically differentiated BM-MSCs. (A–F) Protein and RNA were extracted from group I (cardiomyogenically differentiated BM-MSCs without Ag-NP treatment), group II (BM-MSCs in the presence of 2.5 µg/mL Ag-NPs), and III (cardiomyogenically differentiated BM-MSCs in the presence of 2.5 µg/mL Ag-NPs) as described in methods section and was subjected to western blotting; (*P < 0.05 and **P < 0.01) and real-time PCR (G–K); (*P < 0.05 and **P < 0.01), respectively.

To investigate the effect of Ag-NPs on the cardiomyogenic differentiation of BM-MSCs, the cardiac proteins and genes were examined (Figure 7) [20]. GATA binding protein 4 (GATA4), a cardiac transcription factor, was investigated in this section. The protein and gene expression of GATA4 were significantly increased by factors of about 1.20 and 1.27 times in group III (cardiomyogenically differentiated BM-MSCs with Ag-NPs) as compared to group I (cardiomyogenically differentiated BM-MSCs without Ag-NP treatment), respectively (Figure 7C,H). However, in group II, in the absence of cardiac differentiation medium, Ag-NPs alone did not cause GATA 4 protein expression. Other cardiac differentiation markers, such as cardiac troponin I (C-TnI) (as cardiomyocyte cell marker), smooth muscle actin (SMA) (as smooth muscle cell marker), vascular endothelial growth factor (VEGF) (as vasculogenesis marker), and von Willebrand factor (VWF) (as endothelial cell marker) were investigated in this panel. As shown in Figure 7, no significant increase was seen in the expression of the mentioned cardiac markers. However, the expression of proteins was observed in group II (BM-MSCs with Ag-NPs), in the absence of cardiac differentiation medium. In other words, it could be claimed that Ag-NPs play an important role in the expression of cardiac markers even in the absence of the cardiac differentiation medium.

### Ag-NPs increased the Wnt-3 and β-catenin protein and gene expression

**Figure 8:**
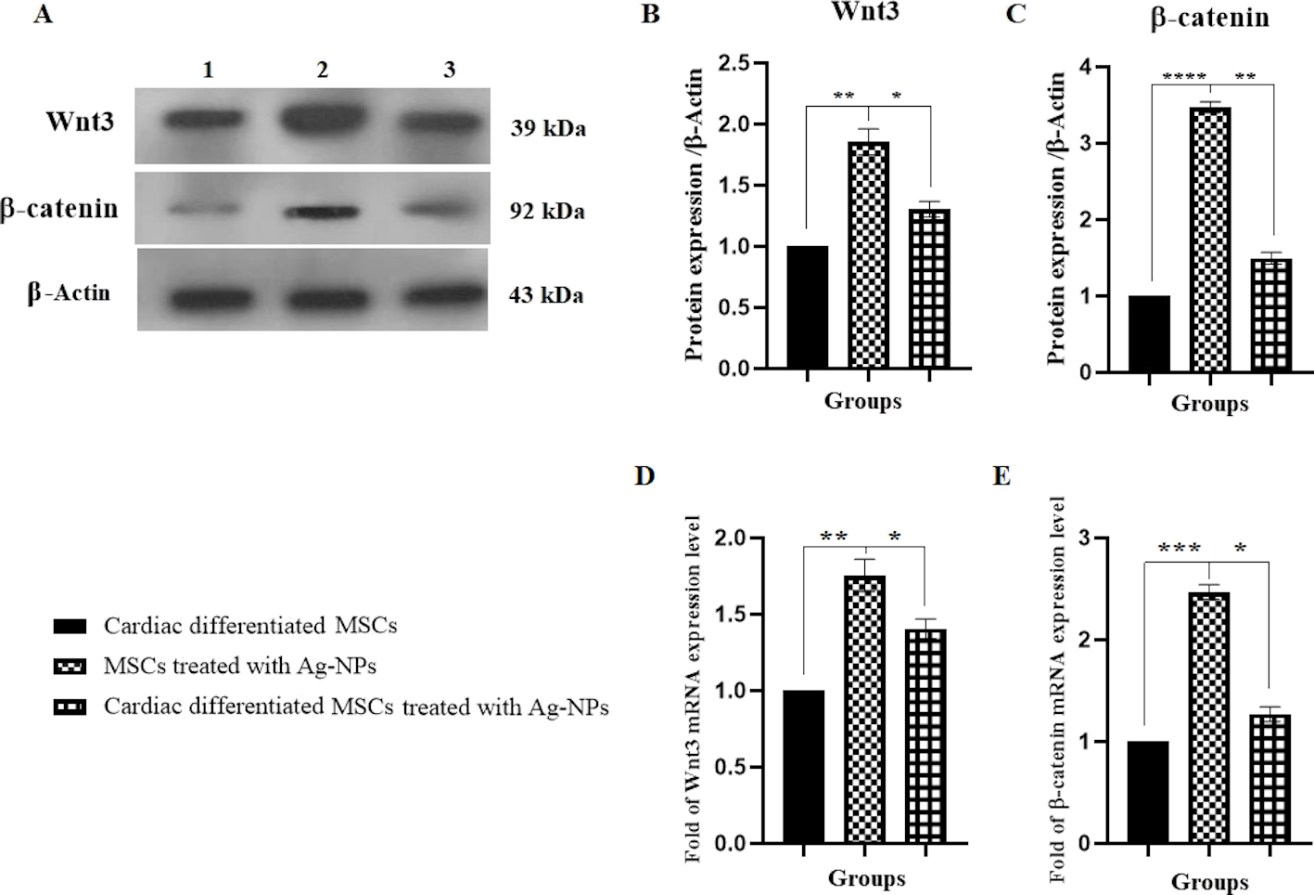
Effect of Ag-NPs on Wnt-3 and β-catenin protein and gene expression in cardiomyogenically differentiated BM-MSCs. (A–C) Protein and RNA were extracted from group I (cardiomyogenically differentiated BM-MSCs without Ag-NP treatment), group II (BM-MSCs in the presence of 2.5 µg/mL Ag-NPs), and III (cardiomyogenically differentiated BM-MSCs in the presence of 2.5 µg/mL Ag-NPs) as described in methods section and was subjected to western blotting; (*P < 0.05, **P < 0.01 and ****P < 0.0001) and real-time PCR (D and E); (*P < 0.05, **P < 0.01, and ***P < 0.001), respectively.

The protein and gene expression levels of Wnt-3 and β-catenin were significantly increased in group II in comparison to group I by about 1.85 and 1.75 (**p < 0.01) and 3.47 and 2.46 (****p < 0.0001 and ***p < 0.001), respectively. Also, a significant increase in the protein and gene expression levels of Wnt-3 and β-catenin were observed in group III as compared to group I by factors of about 1.35 and 1.40 (*p < 0.05) and 1.49 and 1.27 (**p < 0.01 and *p < 0.05), respectively (Figure 8).

## Discussion

MSCs, as multipotent cells, have unique features, such as self-renewal and multilineage differentiation potential, which can be utilized for cell-based therapy [21–22]; however, these features are relatively weak when compared to other stem cells, that is, induced pluripotent stem cells (iPSCs). Meanwhile, MSCs are easier to isolate and expand with fewer technical and ethical problems than other stem cells. For this reason, MSCs have received more attention from researchers [23]. In general, MSCs differentiation is a laborious technique and requires a set of factors, components, and chemicals that initiate the differentiation into cell lineages such as cardiomyocytes, osteocytes, or chondrocytes [24]. Nanotechnology can boost stem cell differentiation and eliminate many obstacles thus improving its applicability in regenerative medicine [25]. The usage of nanomaterials in medicine has been considered previously and subsequent studies should identify relationships between nanomaterials and stem cell differentiation [26]. The potential effects of Ag-NPs on osteogenic and adipogenic differentiation of MSCs have been reported in other studies. In one study by Qin et al., it was shown that 4 µg/mL Ag-NPs could promote the osteogenic differentiation of stem cells through enhancing osteogenic markers of alkaline phosphatase (ALP), runt-related transcription factor 2 (RUNX2), and bone morphogenetic protein 2 (BMP2) [27]. In another study, Zhang et al. indicated that Ag-NPs caused the induction of osteogenic differentiation of MSCs via chemo-attraction of MSCs to migrate to the fracture site as well as activation of transforming the growth factor (TGF)-β/bone morphogenetic protein (BMP) signaling pathway [28]. In contrast to previous reports, He et al. reported that Ag-NPs only increased the adipogenic differentiation of MSCs with the activation of adipogenic markers, not the osteogenic differentiation [29].

In addition to the promotion of osteogenic and adipogenic differentiation by Ag-NPs, the induction of neurogenic differentiation has also been investigated. It was found that Ag-NPs enhanced the neuronal differentiation of stem cells with upregulation of the neuronal markers [30].

The effect of Ag-NPs on the differentiation of MSCs into hepatocyte-like cells and cardiomyocytes was recently investigated by Hu and co-workers [31]. It was pointed that the induction effect of Ag-NPs is concentration-dependent. More specifically, it was shown that 0.1 µg/mL Ag-NPs caused elevated levels of NK2 homeobox 5 (NKX2.5), myosin heavy chain 6 (MYH6), and islet (ISL) genes expression, of alpha-fetoprotein (AFP), albumin (ALB) proteins expression and of hepatocyte nuclear factor 4 alpha (HNF4A) formation as cardiac and liver markers [31]. In the present study, the in vitro effect of Ag-NPs was investigated on cardiac differentiation potency as well as TL of MSCs. The suitable concentration of Ag-NPs which prolong the cell viability of MSCs was determined by MTT assay as previously examined by Sengstock and co-workers [11].

Sengstock et al. reported that Ag-NPs with a size of 80 nm do not enter the cell nucleus and that Ag agglomerates appeared primarily within the endo-lysosomes. In the other words, the agglomerated Ag-NPs were visible in a region close to the cell nucleus but not in the cell culture medium outside the cells [11]. According to a previous study by Fröhlich et al., the access of Ag-NPs into other organelles depends on the particle size [32]. In the same way, Berry et al. demonstrated that the uptake of NPs is limited by the dimensions of the nuclear pores. Gold nanoparticles (Au-NPs) with a size of 5 nm appeared in the nuclei of cells, whereas particles larger than 30 nm were maintained in the cytoplasm [33]. In the present study, we used Ag-NPs with a size of 80 nm, thus, no Ag agglomerates were found in the nucleus.

In addition, aggregated NPs as well as single NPs are present in the solution. The aggregation of NPs depends on the type and amount of ions present in the solutions. In one study, Stebounova et al. concluded that Ag-NPs form aggregates and agglomerates when presented to high ionic fluids. Less than 10 mg/L of Ag-NPs remains suspended, and the rest forms aggregates [34]. Polymer coatings can be used to prevent the NPs from aggregating. In the present study, polyvinylpyrrolidone-coated Ag-NPs were used.

Another important aspect of the behavior of Ag-NPs is the rate and degree of the dissolution of Ag-NPs, which depends on their surface functionalization, their concentration, and the temperature. There is still disagreement about the dissolution behavior of Ag-NPs [35]. In a study by Loza et al., the dissolution of Ag-NPs was monitored using dialysis bags that were permeable only for Ag ions. Various compounds that are present in biological media were added to the immersion medium and dissolution curves were measured [35]. When no oxygen was present, only a very small fraction of Ag was dissolved. In the present of oxidizing agents, such as H_2_O_2_, the dissolution rate was clearly increased. The presence of dissolved NaCl, either in pure form or as PBS, strongly slows down the dissolution [35]. In another study, Zook et al. investigated the dissolution in DMEM cell culture medium and reported the dissolution of different polymer-coated Ag-NPs. It was found that the dissolution was increased in cell culture media in comparison to inorganic salt solutions, which is likely due to complexion of the released silver ions [36].

To explore the effect of Ag-NPs on the cardiomyogenic differentiation of BM-MSCs, we investigated the protein expressions of C-TnI, GATA4, SMA, VEGF, and VWF, as well as those of TERT and cyline D1 in response to Ag-NP treatment. It was shown that in group II (BM-MSCs in the presence of 2.5 µg/mL Ag-NPs), Ag-NPs in the absence of cardiac differentiation medium promoted the protein expression of C-TnI, SMA, VEGF, and VWF. However, this effect was not observed in group III in the presence of cardiomyogenic differentiation medium as compared to the control group (cardiomyogenically differentiated BM-MSCs without Ag-NP treatment). This effect may be due to the fact that Ag-NPs interact with the components of the cardiac differentiation medium and prevent further cardiac differentiation. In other words, we showed that 2.5 µg/mL Ag-NPs can enhance the cardiac differentiation of BM-MSCs through the expression of VEGF and VWF as endothelial cell markers, SMA as smooth muscle cell marker, and troponin T as cardiomyocyte marker in group II. In addition, it was shown that Ag-NPs cause elevated GATA4 protein expression level as cardiac transcription factor in group III as compared to control group. So far, only one study has been performed on determining the role of Ag-NPs and AgNO_3_ on cardiac differentiation of stem cells. In one study by Hu et al., the effect of serial dilution of Ag-NPs was determined on NKX2.5, MYH6, and ISL cardiac markers [31]. The Ag-NPs increased the expression of typical cardiac markers. In addition to the above, cardiac regeneration is mediated by signaling pathways [37]. Wnt3/β-catenin is one of the most important pathways that regulate cardiomyogenic differentiation [37]. For this reason, in another part of this study, in the presence of Ag-NPs, we investigated the protein expression of Wnt3 and β-catenin as a main component in the pathways associated with cardiomyogenesis and TL.

Accordingly, other methods, including use of factors and NPs to dominate cell senescence, will considerably facilitate cell therapy. Telomere biology could be possibly involved in the development of age-associated cardiovascular diseases such as myocardial infarction and heart failure. Shortened telomeres activate a series of downstream changes that induce cardiomyocyte senescence [17]. The reduced proliferative potential of cardiovascular systems limits the regenerative capacity of aged and injured myocardium [38]. Thus, therapeutic strategies to restore the proliferative potential of injured cardiovascular systems are considered as a promising alternative treatment. There is a direct relationship between the TERT protein expression as telomerase catalytic subunit, TL, and telomerase activity [39]. Thus, in addition to the TL, the TERT protein expression was also investigated. It was previously reported that the differentiation efficiency of stem cells into cardiomyocytes correlates positively with the TL. More specifically, it was shown that cells with relatively long telomeres and high expression levels of TERT would differentiate earlier and more efficiently into cardiomyocytes than those with relatively short telomeres and low TERT expression [40]. Aguado et al. indicated that the positive correlation between the efficiency of cardiomyocyte differentiation and TL suggests that the strategies that increase TL in stem cells would augment the yield of cardiomyocytes [40]. Our study indicated that 2.5 µg/mL Ag-NPs can increase the aTL as well as the TERT protein expression of the cardiomyogenically differentiated BM-MSCs. The effect of Ag-NPs was also associated with increased Wnt3 and β-catenin protein expression. Although various studies have been performed on TL and signaling pathways, extensive evaluations are required to demonstrate the exact relationship between TL extension and pathways in the presence of Ag-NPs. For example, the use of inhibitors and/or activators of the Wnt3/β-catenin pathway and re-evaluation of the Ag-NPs effect on TL would provide specific results.

## Conclusion

This research indicated that 2.5 µg/mL Ag-NPs could promote the protein expression of cardiac-related marker GATA-4 of cardiomyogenically differentiated BM-MSCs. Also, there was a significant increase in the protein expression of Wnt3 and β-catenin as a main component of pathways in both groups II and III (BM-MSCs with Ag-NPs and cardiomyogenically differentiated BM-MSCs with Ag-NPs). As an interesting finding, this study showed that 2.5 µg/mL Ag-NPs could elongate the TL and enhance the protein expression of the telomerase catalytic subunit of both groups II and III. This rise was only significant in group II. The effects observed in this study may be related to changes in the protein expression levels of Wnt3 and β-catenin signaling pathway components.

## Experimental

All cell culture plates as well as cell culture media were purchased form SPL Company Ltd. and Gibco Company (UK), respectively. Sources of other materials are mentioned in the text. The Ag-NPs used in this study were produced by an American company (US Research Nanomaterials) and purchased from Iranian Nanomaterial Pioneers Company.

### Ag-NPs structure

**Figure 9:**
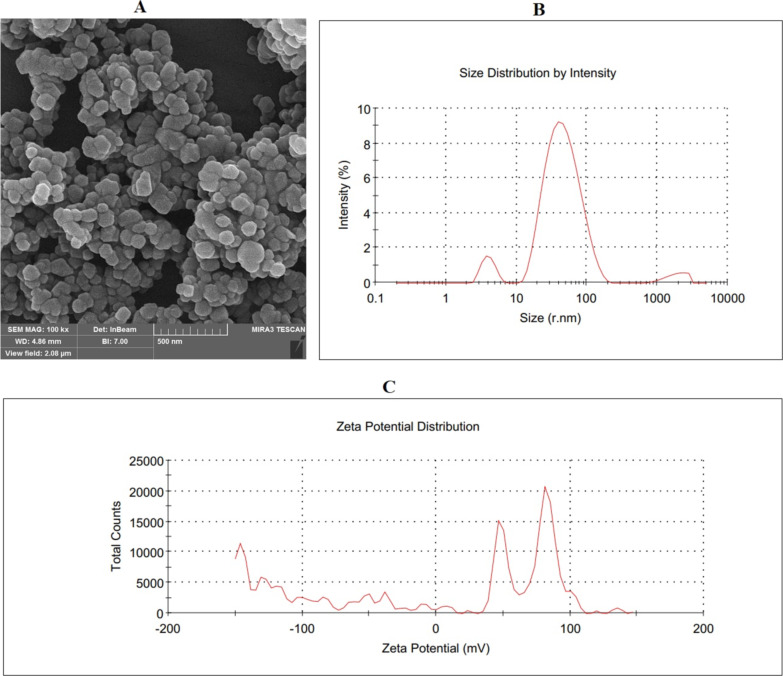
Ag-NPs structure. (A) SEM image of Ag-NPs, (B) size distribution by intensity, and (C) zeta potential distribution.

The Ag-NP powder was dispersed in distilled water without adding surfactants. Ag-NPs with a size of 50–80 nm and 99% purity were used (Table 1). The vendor carried out scanning electron microscopy analysis in the Nano laboratory unit, which is approved by the Iranian Nanotechnology Initiative Council (Figure 9A). Also, the size distribution and zeta potential distribution was provided by the commercial supplier as (Figure 9B,C).

**Table 1:** Ag-NPs characterization.

Purity (%)	APS (nm)	True density (g/cm^3^)	Bulk density (g/cm^3^)	Color	Morphology

99	50–80	10.5	0.35	black	spherical

### BM-MSCs isolation

BM-MSCs were isolated for a previous study by Fathi and co-workers [41]. Briefly, five rats were euthanized using ketamine/xylazine (87/13 mg/kg). After surgery and removal of femur and tibia, BM contents were flushed using washing buffer. Next, the BM contents was layered over Ficoll-Paque (Innotrain, Germany) and mononuclear cells were re-suspended in DMEM supplemented with 10% FBS. The cells were cultured in an incubator until the cells reached 70–80% confluence.

### Characterization of MSCs

Flow cytometry as well as multilineage differentiation were used for the confirmation of BM-MSCs as reported earlier by Fathi and Vietor [42]. For this purpose, 1 × 10^5^ BM-MSCs were incubated with CD31, CD34, CD73, and CD44 antibodies for 30 min at 4 °C. Next, a FACS instrument was used to quantify the florescence intensity of cells. Also, the multilineage differentiation potential was assessed. BM-MSCs were cultured in adipogenic and osteogenic induction medium for 21 days. At the end of induction period, the cells were stained with Oil red O and Alizarin red for adipogenesis and osteogenesis assessment, respectively [43].

### Cardiomyogenic differentiation of MSCs

The cardiomyogenic differentiation method was recently showed by Fathi and co-workers [44]. In brief, 1 × 10^6^ cells/6-well plates were cultured in the cardiomyogenic differentiation medium for 14 days. The medium was refreshed twice weekly. Cardiomyogenic differentiation was confirmed by ICC.

### Immunofluorescence staining

BM-MSCs were seeded in 8-well slide containing cardiomyogenic differentiation medium for 14 days. In the following of cardiomyogenic induction period (14 days), the cells were fixed and were incubated with mAbs against desmin and α-actinin. The cells were then incubated with secondary antibodies for 2 h at 37 °C. Then, cell nuclei were stained with DAPI at a working dilution of 1:1000 for 30 s and the fluorescent cells were visualized under a fluorescence microscope.

### Cell proliferation assay by MTT

The 3-(4,5-dimethylthiazol-2-yl)-2,5-diphenyltetrazolium bromide (MTT) assay measures the mitochondrial activity of the cells [45]. After the BM-MSCs grew to 70–80% confluence, they were washed by PBS and trypsinized with trypsin-EDTA. The 4 × 10^3^ cells were seeded in each well of a 96-well plate with a total volume of 200 μL of cardiomyogenic differentiation medium of different concentrations (0, 1.25, 2.5, and 5 µg/mL) of Ag-NPs and incubated for 14 days at 37 °C in a humidified environment with 5% CO_2_ to grow the cells in a monolayer. The medium was refreshed every three days in each of the experiments.

### Treatment of BM-MSCs with Ag-NPs

Polyvinylpyrrolidone-coated Ag-NPs were purchased from Iranian nanomaterials pioneers (Mashhad City, Khorasan, Iran). The hydrodynamic diameter of the NPs was 50–80 nm as measured by DLS. The suitable concentration of Ag-NPs was obtained by MTT assay. 2.5 µg/mL was used as a final concentration for treatment of BM-MSCs. In this study, the cells were divided into three groups: group I as control group (cardiomyogenically differentiated BM-MSCs without Ag-NPs), group II (BM-MSCs with Ag-NPs), and group III (cardiomyogenically differentiated BM-MSCs with Ag-NPs).

### aTL measurement

Cardiomyogenically differentiated BM-MSCs were divided into three groups: group I (cardiomyogenically differentiated BM-MSCs without Ag-NPs), group II (BM-MSCs with Ag-NPs), and group III (cardiomyogenically differentiated BM-MSCs with Ag-NPs). At the end of treatment time (14 days), genomic DNA was extracted and the absolute TL was measured by real-time PCR as previously described by O’Callaghan and Fenech [46]. The primers used are listed in Table 2 [47].

**Table 2:** Primer sequences used for the aTL measurement.

Oligomer name	Primer pair sequence (5'–3')	PCR product size (bp)

Telomere standard	(TTAGGG)14	84
36B4 standard	5'–CCTTGTCTCCAGTCTTTATCAGCTGCACATCGCTCTGAGGA AGAGAAGAGCAGTTACCACCCAGACACACAGAAG–3'	75
Telo	Fwd: CGGTTTGTTTGGGTTTGGGTTTGGGTTTGGG TTTGGGTT Rev: GGCTTGCCTTACCCTTACCCTTACCC TTACCCTTACCCT	>76
36B4	Fwd: CTTCTGTGTGTCTGGGTGGT Rev: CCTTGTCTCCAGTCTTTATCAG	75

### Gene expression assessment

BM-MSCs were plated at a density of 2 × 10^6^ cells/well in 6-well plates containing cardiomyocyte differentiation medium for 14 days, and were divided into three groups, as described above. At the end of the 14th day, total RNA was extracted and cDNA was synthesized from cardiomyogenically differentiated BM-MSCs in control and experimental groups using molecular kits (Yekta Tajhiz Azma, IRAN). The mRNA expressions of target genes included VEGF, C-TnI, vWF, SMA, GATA-4, TERT, cyclin-D, Wnt-3, and β-catenin. All PCR reactions were performed using a Corbett Rotor-Gene™ 6000 HRM (Corbett Research, Australia). Fluorescence data was calculated in relation to β-actin CT values by the 2^−ΔΔCT^ method. Primers (Table 3) were designed using Oligo 7 v.7.52 software [44,48–49].

**Table 3:** Primer sequences used for the real-time PCR assays.

No.	Gene	Primer pair sequence (5'–3')	Product length (bp)

NM_001204384.1	VEGF	ATCACGAAGTGGTGAAGTTC TGCTGTAGGAAGCTCATCTC	117
XM_006716677.4	C-TnI	GCAGGTGAAGAAGGAGGACA CGATATTCTTGCGCCAGTC	139
NM_000552.4	vWF	GCAGTGGAGAACAGTGGTG GTGGCAGCGGGCAAAC	134
NM_001613.4	SMA	ATCACCAACTGGGACGACAT CATACATGGCTGGGACATTG	175
NM_144730.1	GATA-4	CGGAAGCCCAAGAACCTGA CTGCTGTGCCCGTAGTGAG	179
NM_053423.1	TERT	CAAAAGCCTTTCTCAGCACC CTTAATTGAGGTCCGTCCGT	227
NM_171992.5	cyclin-D	TCGGAACCAGATTCACGTTG AAGGGCATCTGTAAATACACT	169
NM_001107005.2	Wnt3a	ATGTTCGGGACCTATTCCA CTGTAGCATCTCGCTTCC	123
NM_053357.2	β-catenin	CTGTTCTACGCCATCACC TTTCCTGATTGCCGTAAGC	178
NM_001101.5	β-actin	TCCTCTCCCAAGTCCACACAGG GGGCACGAAGGCTCATCATTC	131

### Protein expression assessment

The proteins were extracted from cardiomyogenically differentiated BM-MSCs and each cell protein sample was electrophoresed and transferred to membrane. The membranes were incubated with primary polyclonal antibodies (1:1000) against β-actin (sc-69879), VEGF (sc-7269), C-TnI (sc-133117), VWF (sc-365712), SMA (sc-53015), GATA-4 (sc-25310), TERT (E-AB-33070), cyclin-D (SC-8396), Wnt-3 (sc-74537), and β-catenin (sc-7963), and were incubated with secondary antibody (1:5000). In the following, the protein bands were detected using ECL detection Kit. The intensity of protein bands was measured using the ImageJ 1.6 software [50–52].

### Statistical analysis

The results were analyzed using Graph Pad Prism version 6.01. One-way ANOVA followed by post hoc Tukey's tests was used to determine any significant differences among the groups. Quantitative real-time PCR and flow-cytometry were analyzed using REST 2009 and FlowJo software, respectively. Statistical significance was determined to be *p < 0.05. All experimental procedures were repeated three times.

### Ethical approval

Ethical consent was approved by an ethics committee at Tabriz University of Medical Sciences, Tabriz, Iran (Ethic Code No: IR.TBZMED.VCR.REC.1398.247).
